# ECMO for Adult Respiratory Failure: A Rapid Review of Clinical and Service Delivery Evidence to Guide Policy in Wales

**DOI:** 10.1177/10892532241309787

**Published:** 2024-12-22

**Authors:** Michal Pruski, Michael Beddard, Susan O’Connell, Andrew Champion, Rhys Morris, Richard Pugh, Iolo Doull

**Affiliations:** 1 CEDAR, Department of Medical Physics and Clinical Engineering, University Hospital of Wales, 8903 Cardiff and Vale University Health Board, UK; 2 University of Manchester, UK; 3 645414 NHS Wales Joint Commissioning Committee, Pontypridd, UK; 4 Department of Anaesthetics, 97620 Glan Clwyd Hospital, Bodelwyddan, UK

**Keywords:** ECMO, perfusionist, critical care, centre size, nurse-delivered

## Abstract

**Background:**

While several studies have summarised the clinical effectiveness evidence for extracorporeal membrane oxygenation (ECMO), there are no evidence syntheses of the impact of centres’ ECMO patient volume on patient outcomes or the impact of bedside ECMO care being delivered by either a perfusionist or a nurse. There is also limited information on the cost-effectiveness of ECMO.

**Purpose:**

This review was carried out to evaluate the clinical effectiveness and cost of different service delivery models of pulmonary ECMO to inform NHS Wales commissioning policy.

**Research Design:**

The study utilised rapid review methodology, consisting of a systematic literature search and the inclusion of the highest quality of evidence available.

**Data Collection:**

Out of 1997 records identified via literature searches, 12 studies fell within the scope. The 2 meta-analyses comparing ECMO with lung-protective ventilation favoured ECMO.

**Results:**

Five studies looking at the clinical impact of centre patient volume had large heterogeneity. Three studies estimated that with sufficient patient volume, nurse-delivered ECMO was cost-saving, with thresholds varying between 92 and 155 patient days per year. Three studies looked at the cost impact of ECMO delivery, with ECMO being cost incurring, but potentially cost-effective, with costs per patient being lower at higher volume centres.

**Conclusions:**

The available evidence supports the use of ECMO in adult respiratory failure patients, despite it being cost-incurring. ECMO can be nurse-delivered without a significant negative impact on patient care. Yet decision-makers need to consider their local circumstances when making commissioning decisions.

## Introduction

Respiratory failure has various physiological aetiologies including ventilation-perfusion mismatch, anatomical shunting, diffusion limitation and hypoventilation.^1^ In cases of acute respiratory distress syndrome (ARDS), the recommended treatment is lung-protective mechanical ventilation, characterised by low tidal volumes and low plateau pressures.^2^ These aim to minimise lung trauma due to overdistension but are not always effective at ensuring adequate blood oxygenation and carbon dioxide removal. Additionally, adjunctive strategies can be employed to optimise respiratory care in moderate to severe ARDS if clinically indicated.^2^

Extracorporeal membrane oxygenation (ECMO) is a highly invasive procedure that takes over the function of the lungs (and potentially the heart), bypassing the physiological limitations of gas exchange that inhibit the effectiveness of mechanical ventilation in such conditions as ARDS. ECMO may also be used in contexts of cardiogenic shock, as a bridge to heart or lung transplantation, and in the context of cardiopulmonary resuscitation. ECMO can be set up so that either the returned blood enters the patient’s artery (veno-arterial ECMO) or into their vein (veno-venous ECMO). The Extracorporeal Life Support Organization’s (ELSO) registry (as of 26 September 2024) has 140,204 adult ECMO cases recorded, 60,218 for pulmonary indications, 60,986 for cardiac indications and 19,000 for cardiopulmonary resuscitation.^3^ For pulmonary indications the majority of cases utilised veno-venous ECMO (54,804), while for the other 2 indications veno-arterial ECMO was the predominant modality.^3^ National Health Service (NHS) England registry data indicates that ECMO has increasingly been used in this setting when conventional measures, such as lung-protective mechanical ventilation used together with adjunctive therapies,^2^ are inadequate.^4^ Moreover, this NHS England registry data shows a survival rate of 74% at ECMO intensive care unit (ICU) discharge.^4^ A recent NHS consensus NHS document defined criteria to support decision-making regarding ECMO when such adjuncts prove inadequate.^5^

The COVID-19 pandemic greatly tested the ability of ICUs to deal with severe respiratory failure. Consequently, while the 2020 World Health Organization (WHO) guidance on COVID-19 management referred to the use of ECMO in COVID-19 patient care,^6^ a recent systematic review reported 49% mortality among ECMO COVID-19 patients, with predictors of mortality including age, adjunctive use of steroids, and pandemic phase.^7^

ECMO is resource-demanding, requiring a high degree of technical skill, and is associated with risks such as bleeding, thrombosis, renal failure, neurological injury and infection.^8^ ECMO use is also associated with a decrement in health-related quality of life compared to survivors of conventional mechanical ventilation,^9,10^ though some of these risks might be the consequence of the underlying pathology. Consequently, ECMO patients require constant support from clinical perfusion scientists and/or suitably qualified ECMO nurses.

In the UK there are a limited number of specialist centres delivering ECMO ensuring an appropriate standard of service delivery.^11,12^ Hence, commissioning for respiratory ECMO and patient retrieval is done nationally. Guidance on the provision of ECMO services is available from both NHS England and the National Institute for Health and Care Excellence (NICE).^11-13^ International guidance on the use of ECMO in adults with respiratory failure is available from the ELSO.^14^

### Justification for the Review

The Welsh Health Specialised Services Committee (WHSSC), which as of 1 April 2024 has been subsumed into the NHS Wales Joint Commissioning Committee, commissioned specialised services for the population of Wales predicated on evidence-based policies. WHSSC required a rapid review of the evidence basis for the use of ECMO in adult respiratory failure, to guide commissioning decisions. WHSSC required information on:

- Evidence on the clinical effectiveness of ECMO for respiratory failure in adults compared to lung-protective mechanical ventilation (with or without the use of adjuncts).
- Evidence regarding various models of ECMO service delivery including the impact of centre patient volume on patient outcomes, and the effect of nurse-delivered vs clinical perfusion scientist-delivered ECMO patient care on patient outcomes and costs (NB: by ‘delivered’ we refer to the profession that provides the majority of the day-to-day management of the ECMO pump and circuit).
- Economic data regarding respiratory ECMO provision.

This publication summarises the policy guidance review used by WHSSC in its decision-making. Given the focus of the review, when the term ‘ECMO’ is used without other clarification it refers to pulmonary ECMO, which encompasses mostly veno-venous ECMO, but may also include veno-arterial ECMO in cases of mixed cardiopulmonary failure.

## Methods

The aim of this evidence review was to support clinical commissioning decisions for ECMO services in Wales in a timely manner. Rapid review methods were employed, whereby comprehensive search strategies were developed and a pragmatic approach to evidence selection was employed. Such a pragmatic rapid review methodology approach is common in health technology assessments.^15^ The search strategy (Supplemental File 1) was designed based on the review scope (supplementary file 2) and run in Medline ALL (Ovid). The strategy restricted the searches to studies conducted after 2000 to account for the emergence of a trend to utilise lung-protective ventilation strategies (such as low tidal volume ventilation) which would have potentially affected the ECMO comparator treatments. The search strategy was then adapted for searches using Embase (Ovid), the Cochrane Database of Systematic Reviews, the Cochrane Central Register of Controlled Trials, and the International HTA database. Searches were conducted between 23^rd^ September 2021 and 7^th^ October 2021. Supplementary searching was also employed using a generic search engine and Google Scholar during the scoping phase of the project, and additional studies were sought by consulting guidelines and snowballing of reference lists of included studies.

Records potentially meeting the inclusion criteria (Supplemental File 2) were screened by 2 reviewers, first at title/abstract stage, then at full text stage. Narrative reviews and non-comparative studies were excluded. Any studies excluded at full text were recorded within a separate file with reasons for exclusion (Supplemental File 3). Critical appraisal of the included meta-analysis studies was carried out by using the AMSTAR2 (revised version of A MeaSurement Tool to Assess systematic Reviews) and TSD7 (NICE DSU Technical Support Document 7: Evidence Synthesis of Treatment Efficacy in Decision Making: A Reviewer’s Checklist) tools and checked by a second reviewer.^16,17^

Data on clinical effectiveness, cost-effectiveness, and safety were extracted from each publication into an evidence table. The limitations of the included studies were noted by 1 reviewer and checked by a second reviewer. The evidence regarding the clinical effectiveness, cost-effectiveness and safety of each intervention were summarised narratively. Subgroup analysis was beyond the scope of this study (Supplemental File 2).^18^

## Results

### Search Results

**Figure 1.**
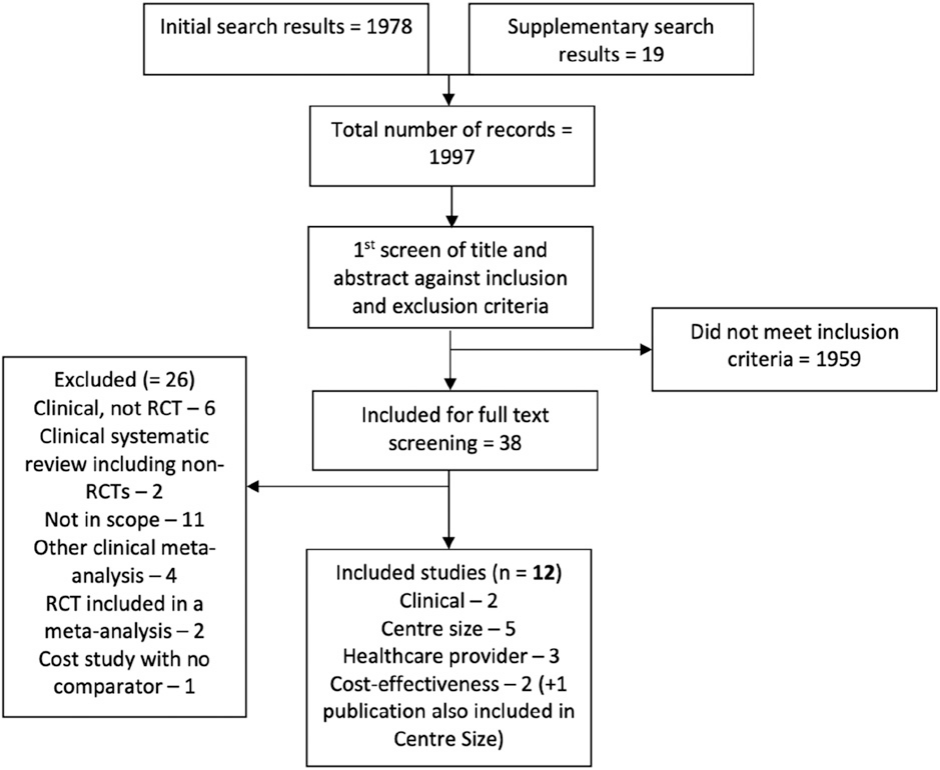
Flow diagram of studies. Adapted from PRISMA.^19^

A total of 1997 studies were identified following removal of duplicates (Figure 1). Of these, 38 were screened at full text. Due to the number of studies identified and their quality, the review team decided to include only systematic reviews/meta-analyses where possible. This decision resulted in a total of 12 studies including; two meta-analyses looking at clinical effectiveness of ECMO; 5 studies looking at the effect of centre size on ECMO care outcomes; 3 studies comparing clinical perfusion scientist and nurse-delivered ECMO care, and 3 publications looking at the cost-effectiveness of ECMO.

The only 2 randomised controlled trials (RCTs) were included in the identified meta-analyses, so were not individually included at the data extraction stage. The limitations of these studies have been discussed below. To avoid duplication of results, only 2 of the identified meta-analyses were included, which utilised different methodologies to analyse the studies. The remaining clinical studies (non-RCTs) were not included since ECMO patients receive complex care and as such it is unlikely that these studies would offer good comparability between the treatment cohorts.

### Clinical Effectiveness

Three studies were noteworthy: 2 network meta-analyses of a variety of respiratory support interventions in ARDS and 1 patient-level meta-analysis of the only 2 RCTs, the CESAR and EOLIA trials, comparing ECMO to conventional ventilation. Table 1 provides a summary of the 2 included studies, with more detailed information presented in supplementary file 4.

**Table 1.** Summary of Key Information From the Included Clinical Studies Comparing ECMO to Mechanical Ventilation.

Study	Study Design	Patient Population	Study and Patient Numbers	Key Outcome
Aoyama et al. 2019^18^	Network meta-analysis of RCTs or quasi-RCTs	Adult patients with moderate to severe ARDS	Total studies = 25 total patients = 7753	28-day mortality network risk ratio (95% credible interval): Lung-protective ventilation vs ECMO 0.60 (0.38–0.93; favours ECMO)
Combes et al.^21^ 2020	Patient-level meta-analysis	Adult patients with ARDS fulfilling the American–European consensus Conference definition or the Berlin definition for ARDS.^24,25^	Total studies = 2 (both also included in Aoyama et al.) total patients = 429	90-day mortality relative risk (95% confidence interval): 0.75 (0.6–0.94; favours ECMO)

There was a high degree of overlap between the 2 network meta-analyses and both studies were judged to be of similar quality using the TSD7 tool.^17^ While Aoyama et al. lacked detail in its assessment of study heterogeneity and of potential inconsistencies in the network, the Sud et al. search strategy was of limited quality and there was a lack of consistent justification for the measurement scale chosen.^18,20^ The decision to include Aoyama et al. while excluding Sud et al. was because Aoyama et al. considered the 2 RCTs (CESAR and EOLIA) as utilising lung-protective ventilation in their control groups which was consistent with our predefined review scope (supplementary file 2). Sud et al. only considered the EOLIA trial as utilising lung-protective ventilation in its control group and did not include the CESAR study.^18,20-23^ Aoyama et al. included 25 studies of patients with moderate to severe ARDS, comparing lung-protective ventilation on its own to ECMO (2 of the 25 studies investigated ECMO) and a variety of adjunctive interventions.^18^ The primary outcome was 28-day mortality, while the secondary outcome of interest was barotrauma. ECMO in severe ARDS cases compared favourably to lung-protective ventilation (direct evidence of moderate quality; risk ratio 0.6 with a 95% credibility interval of 0.38–0.93). There were no significant differences between interventions in the risk of barotrauma, with variable quality of evidence.

The patient-level meta-analysis by Combes et al,^21^ utilises data from the CESAR and EOLIA trials which compared ECMO with lung-protective mechanical ventilation in patients with severe ARDS.^21-23^ The CESAR trial took a ‘pragmatic’ approach, only advising lung-protective ventilation, rather than mandating it, while the EOLIA trial had a strict ventilation protocol.^22,23^ Thus, 70% of control group patients in the CESAR trial received lung-protective ventilation, as opposed to all patients in the EOLIA trial. Both trials allowed for adjunct therapies, such as those evaluated in Aoyama,^18^ to be used at the discretion of the treating physician. The main outcome assessed in Coombes et al. was 90-day mortality, which was 36% in the ECMO group and 48% in the conventional ventilation group and a relative risk of 0.75 (95% CI 0.6–0.94; *P* = 0.013). Lung-protective ventilation was received by 98% of patients in the ECMO group and 85% of patients in the conventional ventilation group.

### Centre Size

The evidence from 5 studies assessing the impact of centre size (as defined by the number of patients treated) on patient outcomes in a variety of geographical locations is briefly summarised in Table 2. Centre size impact was assessed to see if the evidence suggests whether ECMO should be delivered by many smaller centres or a few larger centres. Four studies considered in-hospital mortality as their main outcome measure,^26-29^ while 1 gave information on patient survival to ECMO weaning, ICU discharge and hospital discharge.^30^ 4 studies looked specifically at the correlation between centre size and in-hospital mortality in wide geographical regions,^26-29^ while 1 single centre study assessed the effects of the implementation of a new service delivery model associated with an increased case load.^30^ The studies used different data analysis approaches, including how they defined centre size and the groups of patients for which they conducted secondary analysis. Of note, 2 studies highlighted the importance of the year 2008 for service provision due to ECMO technology advances and increase in ECMO use that occurred as an aftermath of the H1N1 pandemic.^26,28^

**Table 2.** Summary of Key Information From Studies Comparing Centre Size and Patient Mortality. Where Available, Data Most Pertinent to Respiratory Failure Patients Has Been Presented.

Study Details	Sample Size, n	In-Hospital Mortality Odds Ratio (95% CI)
Bailey et al. (2018)^27^ USA	Low volume: 2548 cases over 322 institutions^a^ Medium volume: 5278 cases over 328 institutions^a^ High volume: 10 585 cases^a^ over 319 institutions	Low-volume: Reference Medium-volume hospital: 1.25 (0.97–1.61) High-volume hospital: 1.61 (1.25–2.07) (Whole study population data)
Barbaro et al. (2015)^28^ world-wide	Veno-venous ECMO: 2377 Respiratory support: 2754	0.99 (0.86–1.15) 0.95 (0.83–1.108) (Case load from 2008–2013 treated as a continuous variable, with model evaluating an increase of 30 cases while controlling for other variables)
McCaffrey et al. (2016)^30^ Australia	Old model: Median <5 cases per year New model: Median = 10 cases per year	Old model: 50.0%^b^ New model: 26.7%^b^ (survival data from the veno-venous ECMO subpopulation)
McCarthy et al. (2016)^26^ USA	Mean 2 cases per year:^c^ 467 Mean 7 cases per year:^c^ 285 Mean 19 cases per year:^c^ 445	Low volume compared to high volume: 1.2 (0.65–2.1) Medium volume compared to high volume: 2.1 (0.95–4.5) (Multivariable model for respiratory failure patients)
Muguruma et al. (2020)^29^ Japan	<8 cases per year: 400 8–16 cases per year: 419 >16 cases per year: 458	<8 cases per year: Reference 8–16 cases per year: 0.72 (0.50–1.04) >16 cases per year: 0.65 (0.45–0.95) (Data pertains only to respiratory failure ECMO patients)

^a^It is unclear if each institution contributed patients over all years.

^b^Calculated based on the study’s patient survival values; the values were the same for ECMO weaning, ICU survival, and hospital survival endpoints.

^c^Calculated by authors from the data presented in the study. Please note that there was high variability in the tercile cut-off scores used annually; as examples of the variability the approximate cut-off values in the first and last year (estimated from a figure in the original publication) of the study were 1, 2, and 4 patients per year vs 12, 47, and 58 patients per year.

NB: As Barbaro et al^28^ utilised the ELSO registry, it is possible that it includes data from the centres involved in the other 4 studies, and that consequently data from some patients has been included in more than 1 study.

Using terciles to define centre size and similar methodology utilising the US National Inpatient Sample (NIS), McCarthy et al. and Bailey et al, 2 studies conducted in the USA, reported that low-volume centres had the best patient outcomes.^26,27^ McCarthy et al. assessed discharges within the NIS of adult patients who underwent ECMO between 2002 and 2011.^26^ Both unadjusted in-hospital mortality rates for all ECMO admissions and survival to discharge were significantly better for low-volume institutions (*P* < 0.01 in both cases). A subanalysis for respiratory failure patients showed that unadjusted mortality rates were 47% in low-volume centres, 61% in medium-volume centres and 56% in high-volume centres. There was a significance difference between low and medium-volume centres (*P* = 0.045), but not between low and high-volume centres (*P* = 0.15). A multivariate analysis of adjusted mortality did not show any significant difference between all terciles.^26^ Bailey et al also used the NIS to explore the relationship between institutional discharge volume and patient outcomes between 2008 and 2014.^27^ Unadjusted mortality at low-volume centres was less than that of medium (43.7% vs 50.3%, *P* = 0.03) and high-volume centres (43.7% vs 55.6%, *P* < 0.001). Respiratory failure as an indication for ECMO was an independent predictor of mortality (OR 1.81, *P* < 0.001), while exclusion of transferred patients from analysis still demonstrated a higher mortality in high volume compared to low-volume centres (50.2% vs 42.8%, *P* = 0.004). Importantly, only 35% of ECMO patients were treated for respiratory failure in low-volume centres, while 45% of ECMO patients were treated for this indication in high-volume centres. There were however differences in the definition of centre size – McCarthy stratified centre size based on the annual number of ECMO cases performed at the centre while Bailey stratified centre size on total annual patient discharges (not just ECMO cases).^26,27^

The world-wide study by Barbaro et al. reported that adult patients who received ECMO for any indication between 2008 and 2013 in centres that treated at least 15 patients per year had better mortality outcomes than those treating less than 6 patients per year (table 2).^28^ This better performance of higher volume centres remained true when the centre volume was treated as a continuous variable (*P* < 0.001). Sensitivity analysis highlighted that this advantage primarily applied to veno-arterial ECMO, (*P* < 0.001) and to patients receiving ECMO for cardiac support (*P* = 0.004). There was no statistically significant difference in outcomes by centre size for veno-venous ECMO patients (*P* = 0.91) or when ECMO was used for respiratory support (*P* = 0.42).^28^

Muguruma et al. looked at respiratory failure ECMO patients, which represented 5% of all Japanese ECMO cases. Their primary analysis showed that Japanese centres treating at least 17 patients a year had lower mortality (*P* = 0.024) compared to centres treating less than 8 patients annually, although it is unclear if centre size was calculated by respiratory failure ECMO case load or all ECMO case load.^29^ Secondary analysis utilising the same cut-offs as in Barbaro et al^28^ showed that a significant improvement in patient mortality, compared to centres treating less than 6 patients a year, only existed for centres treating 15–30 patients a year (*P* = 0.016) but not for centres treating more than 30 patients per year.^29^

McCaffrey et al. found no difference in in-hospital survival before and after the implementation of a new ECMO clinical service model in an Australian centre, which was associated with an increase in annual patient volume.^30^ Across their two cohorts, 21 out of 61 patients received veno-venous ECMO. They identified a decrease in mechanical (*P* = 0.02) and cardiovascular (*P* = 0.02) complications, but not in other types of complications with the new service delivery model. Nevertheless, the results of this study should be considered with caution as it was not designed to assess the impact of yearly patient case load on patient outcomes. Moreover, the change in service delivery models would have introduced additional confounders into the data analysis.

### Health care professional delivery of ECMO

The primary evidence on models of health care professionals delivering ECMO patient care comes from 3 studies summarised in Table 3. ECMO care has been historically delivered by clinical perfusion scientists, and the aim of this part of the review was to elucidate if ECMO care could be delivered primarily by trained nurses without a negative effect on patient outcomes. Two of the 3 studies reported survival to discharge,^31,32^ and 1 reported hospital mortality.^33^ All 3 studies are set in the USA and are retrospective comparisons of ECMO care delivered by clinical perfusion scientists or nurses. None of these studies provided a subanalysis of outcomes for respiratory failure ECMO patients, and less than half of these patients (Table 3) were likely to be treated for respiratory indications. Nevertheless, it was assumed that bedside ECMO management would be similar for cardiac and respiratory indication patients. In all 3 studies ECMO care was led by physicians or surgeons and initially delivered at the bedside by clinical perfusion scientists and in later years moved to a nurse-delivered model. The way nurse-delivered programmes were implemented varied between the studies. Dhamija and colleagues evaluated this service for a range of patients receiving veno-venous and veno-arterial ECMO, where the clinical perfusion scientists provided ECMO care for the first 24 hours, which was then taken over by a nurse or respiratory therapist, with the clinical perfusion scientist being available on call.^31^ Odish et al. does not mention any clinical perfusion scientist support to the ECMO nurse.^32^ Neither of these studies mentions if the ECMO nurse replaced the general ICU nurse or if the ECMO nurse was in addition to the ICU nurse. Cavarocchi and colleagues^33^ explicitly mention it was the general ICU nurses that undertook ECMO care in their study, with the clinical perfusion scientist and intensivist being on call to respond to any problems.

**Table 3.** Summary of Patient Survival to Discharge Data in Studies Comparing Perfusionist- and Nurse-Led Programmes.

Study Details	Intervention Group	Sample Size (n) (% of most relevant indication)^b^	Survival to Discharge, n (%)	Patient volume at which nurse volume becomes more cost-effective
Odish et al. (2021)^32^ USA	Perfusionist-delivered ECMO	29 (24.1%)	8/29 (27.5)	155 patient ECMO days annually
	Nurse-delivered ECMO	94 (38.3%)	49/94 (52)	
Dhamija et al. (2021)^31^ USA	Perfusionist-delivered ECMO	77 (53.2%)	48/77 (62.3)	ECMO mean duration of >9.7 days per case OR ECMO case volume exceeding 10 cases/year
	Nurse-delivered ECMO	24 (37.5%)	14/24 (58.3)	
Cavarocchi et al^33^ (2015) USA	Perfusionist-delivered ECMO	28 (25%)	16/28 (57)^a^	1 patient/month on ECMO for 7.7 days or more
	ICU/Nurse-delivered ECMO	46 (20%)	19/46 (41)^a^	

^a^Survival to discharge was not reported in Cavarocchi et al.^33^ and so mortality figures were back-calculated to assume the survival to discharge figure.

^b^In Odish et al.,^32^ this was patients treated for hypoxia and hypercapnia, for Dhamija et al.^31^ it was veno-venous ECMO patients, and for Cavarocchi et al.,^33^ it was patients with ARDS.

All 3 studies reported no statistically significant differences in patient survival/mortality between nurse-delivered and clinical perfusion scientist-delivered ECMO care,^31-33^ although only Odish et al. included non-inferiority in their study design.^32^ Other outcomes reported in the studies included the number of ECMO days, complications, ICU length of stay and hospital length of stay, but no statistically significant differences between the groups were observed. Therefore, these studies suggest that nurse-delivered ECMO care is not associated with significantly different outcomes compared to clinical perfusion scientist-delivered care.

All studies looked at the financial implications of utilising a nurse-delivered programme.^31-33^ Odish et al. found that it would be cost neutral to maintain a nurse-run program with only 155 patient ECMO days annually, with any additional days leading to cost-savings.^32^ Dhamija and colleagues modelled 4 scenarios ranging between 5 and 50 ECMO cases annually, stating that ‘[a]t low utilization there was a near-negligible cost difference between the perfusionist-led model and the nurse-led model’.^31^ The nurse-delivered model became cost beneficial once case load increased beyond 10 ECMO cases per year, or when the average ECMO duration was greater than 9.7 days per patient.^31^ Cavarocchi et al^33^ identified the financial breakeven point to occur when 1 patient was treated on ECMO for 7.7 days every month. As such, nurse-delivered programmes are suggested to be cost-saving when annual patient ECMO days reached between 92 and 155 days.

### Cost and Cost-Effectiveness

A systematic review of ECMO cost studies was undertaken by Oude Lansink-Hartgring et al, but the only comparative study they identified was the CESAR trial.^22,34^ The CESAR trial found that allocation to ECMO was associated with a mean gain of 0.16 quality-adjusted life years (QALYs) at 6 months after randomisation compared with conventional management.^22^ Consequently, while the cost of ECMO is more than twice the cost of using mechanical lung ventilation in severe ARDS cases, the lifetime predicted cost-utility of ECMO is about £19,000 ($31,000) per QALY, which the authors state at 2005 price levels.^22^ The CESAR study authors included transfer costs as well as the cost of relative visits in their analysis, but gave no indication of the extent to which transfer costs had an impact on the overall costs.^35^

Bailey et al. undertook a propensity-matched analysis showing that higher centre volume is associated with lower costs per patient (*P* < 0.001).^27^ They have also shown that transferred patients incurred higher costs than non-transferred patients, with mean costs at high-volume institutions being $190,299 ± 172,143 for transferred patients vs $168,970 ± 143,954 for non-transferred patient (*P* = 0.009). Bailey et al. also showed that 38.5% of high-volume institution patients were transferred from other acute care centres. Propensity-matched analysis suggests that when transferred patients were excluded, medium-volume centres had the highest cost per patient ($159,607 ± 164,621), but there was no direct comparison made between medium and low-volume centres. The authors did not present a subanalysis for patients receiving ECMO for pulmonary indications. Nevertheless, as noted earlier, higher volume centres had the largest proportion of ECMO patients treated for respiratory failure out of the 3 volume groups in this study.^27^

Nguyen et al. analysed a subset of COVID-19 patients with ARDS who were managed with or without ECMO.^36^ In a simple cost analysis, the mean direct cost of hospitalisation in the ECMO group was $138,403 ± 99,173, vs $48,419 ± 44,799 for the group without ECMO (*P* < 0.01), making ECMO cost-incurring.^36^ The authors only stated that they included direct costs, but did not give more detail.

## Discussion

### Clinical Effectiveness

Both the meta-analysis reported by Aoyama et al. and Combes et al. showed that in the treatment of patients with severe ARDS refractory to conventional treatment, ECMO confers a survival benefit compared to mechanical ventilation.^18,21^ Importantly, while the stated scope of Aoyama et al. was broader than severe ARDS, the ECMO studies it included focused exclusively on patients with severe ARDS. As such, the evidence for the clinical benefit of ECMO over mechanical ventilation cannot be extended to all patients receiving ECMO for respiratory failure.

### Centre Size

The variability in the data concerning the effect of centre size on ECMO patient outcomes presents the greatest challenge in reaching a conclusion as to the optimal model of ECMO service delivery. Most notably, the included studies employed various definitions of centre size categories, but there are also study-specific issues which compound this problem. In Bailey et al. there were notable differences in the patient case-mix, where the majority of patients in low-volume centres received ECMO for post-cardiotomy syndrome and such patients might have better outcomes due to pre-surgical optimisation, while in high-volume centres respiratory failure was the main reason for receiving ECMO.^27^ This indicates that centres of different sizes cared for patients of varying complexities and prognosis. Consequently, an important confounder might be at play, potentially masking the benefit conferred by treatment in larger centres.

The findings of McCarthy et al. are hampered by the significant change in the provision of ECMO over the course of the study – the number of institutions performing ECMO increased from 22 in 2002 to 56 in 2011, with the largest increase occurring post-2008 (after the N1HI pandemic).^26^ Consequently, the mean number of cases performed within each tercile changed over the course of the study. In the case of McCaffery et al, their results should be interpreted with particular caution due to the differences in care received by the study cohorts consequent to its before-and-after design.^30^ While Barbaro et al. found that larger centres had better patient ECMO outcomes, centre size did not have an effect on the outcomes of patients receiving ECMO for respiratory indications in their sensitivity analysis.^28^

Muguruma et al. is the only study which focused on respiratory failure ECMO patient outcomes and found in their primary analysis that high-volume centres performed better than low-volume centres, although their secondary analysis suggest that this relationship stops for centres with over 30 cases a year. Whether centres of any particular size were more likely to take patients with poorer prognosis is uncertain from the information presented in Muguruma et al.^29^

The most likely interpretation of the data, is that larger centre size is associated with improved patient outcomes, but that the largest of centres also receive more complex patients, which would result in the largest centres not having the best overall outcomes, unless patient outcomes were adjusted to take account of patient complexity. Such a masking effect of these more complex patients could affect the largest centres, while sparing the second largest grouping of centres, with such centres benefiting from centre size expertise but being protected from receiving the most complex patients. Because cardiac ECMO patients might be better optimised before receiving ECMO, this effect might not manifest itself as readily in the cardiac ECMO population. This could explain why Barbaro et al, who in their sensitivity analysis looked at a caseload difference of 30 cases, did not find a correlation between centre size and respiratory ECMO outcomes, as well as why Muguruma et al. found centres with a caseload 15 to 30, and not the largest centres, to have the best performance.^28,29^

There are additional reasons which support this interpretation of the data advocating the use of high-volume centres. ELSO and The International ECMO Network (ECMONet) have released position statements on adult ECMO programmes for both acute respiratory failure and cardiac failure, which highlight that centres should have a volume of at least 20 and 30 cases per year, respectively, for both indications.^37,38^ Moreover, both statements highlight the importance of the expertise offered by high-volume centres.^37,38^ This shows that there is strong consensus among experts about the benefit of large volume centres. Similarly, data from the paediatric population, suggest that ECMO centre volume should be at least 22 cases per year.^39^ This preference for treatment in specialist ECMO centre was also reflected in the CESAR trial, where patients were preferentially transferred to such centres.^22^ Lastly, Barbaro et al. proposed a theoretical model which could explain why an improvement in patient outcomes would be expected from an increased in centre case load.^28^ As such, the available data, in conjunction with expert opinion and theoretical considerations, supports the use of centres with higher patient volumes.

### Health care Professional Delivery of ECMO

When considering the available data alongside the NHS pay scale, it appears that nurse-delivered care is likely to be less costly in the UK. Although the identified studies were conducted in the USA, in the UK, qualified clinical perfusion scientists typically enter the workforce at Agenda for Change Band 7, with many employed at Band 8a. In contrast, ECMO bedside nurses are usually employed at Band 5 or 6, and specialist ECMO nurses at Band 6 or 7. This suggests that a nurse-delivered ECMO service in the UK has the potential to also be cost-saving compared to a perfusionist-delivered service.^40^ Moreover, nurse-delivered ECMO programs are likely to be associated with increased ECMO capacity due to workforce availability.^32^ However, the implementation of such staffing strategies must utilise an appropriate governance framework to ensure that reductions in ECMO costs are accompanied by the maintenance of patient safety.^41^ While there was heterogeneity in the specific staffing models employed among the included studies, the fact that they reached similar conclusions broadens their applicability. Nevertheless, these studies were not limited to patients receiving ECMO only for respiratory indications, and as such, there is some uncertainty as to whether the staffing models affect patients receiving ECMO for various indications differently.

### Cost and Cost-Effectiveness

The evidence indicates that ECMO is cost-incurring compared to mechanical ventilation, yet findings from the CESAR trial suggest that it is likely to be cost-effective.^22^ Both the CESAR trial and Bailey et al. suggest that larger centres are associated with lower ECMO costs.^22,27^ Though having more, albeit smaller sized centres, would reduce transport costs, which is a significant contributor to ECMO care expenses.^22,27^ Importantly, while transfer costs could be seen as a confounder to the ECMO cost itself, it is an important consideration when evaluating the impact service provision models on patients and their families.

### Limitations

This rapid review was designed to provide policymakers in Wales, UK, with key evidence pertaining to respiratory ECMO to aid them in decision-making. As such, while we did utilise a systematic search of the academic literature, we did not undertake an in-depth search of the grey literature, thought we did look at guidance from key organisations, such as the NHS, ELSO and WHO. We only included meta-analysis in our review of the clinical effectiveness of ECMO, which only included 2 ECMO RCTs. To avoid duplication of results, only 2 of the identified meta-analyses were included, which utilised different methodologies to analyse the studies. The remaining clinical studies (non-RCTs) were not included since ECMO patients receive complex care and as such it is unlikely that these studies would offer good comparability between the treatment cohorts. Because of these selection criteria, there is a potential that we have missed some relevant information to ECMO’s effectiveness, but we did present the most reliable information. Moreover, we did not consider some of the studies that looked at more focused groups of respiratory failure patients.^42-44^

Our study is also limited by the quality of the underlying evidence. There has been debate surrounding both the CESAR and EOLIA trials, and the degree to which they represent the potential benefit from ECMO itself or from treatment being delivered by a centre specialising in the care of patients with severe respiratory failure. The evidence looking at the impact of ECMO centre size and of nurse-delivered vs clinical perfusion scientist-delivered bedside ECMO care was often not specific to respiratory indications. Nevertheless, if respiratory ECMO is to be delivered in a centre where cardiac ECMO is also delivered, then the presented evidence is highly relevant. Finally, there was limited comparative data on the costs of ECMO vs mechanical ventilation, with the only comparative study identified being the CESAR trial.^22,34^ As such, while more comprehensive studies of ECMO costs are available, none offer more comparative data.

## Implications for Further Research

The primary limitation of this study, given its objectives, is the uncertainty regarding the applicability of the data to the Welsh population. While CESAR was a UK based study, there are many unique characteristics specific to Wales, including its geography and NHS organisation, which may restrict the generalisability of English data. These factors include extensive rural areas and a concentration of large hospital centres primarily situated on the South coast.

There is still a lot of uncertainty in ECMO studies. While the CESAR trial supported ECMO’s effectiveness,^22^ EOLIA did not find ECMO to confer a survival benefit.^23^ Nevertheless, a Bayesian re-analysis of the data from the EOLIA trial, assessing the same end points, corroborates CESAR’s conclusion that ECMO confers clinical benefit to the ARDS population.^45^ Although, as highlighted in the ‘Limitations’ section, there is some debate as to whether CESAR’s findings pertain more to the benefit of ECMO or patient care in a high-volume specialist respiratory unit. As such, there is still uncertainty regarding the clinical benefit of ECMO in the literature, despite its growing clinical adoption, and little is known about its current cost-effectiveness.

While the data comparing bedside ECMO delivery by nurses and clinical perfusion scientists is unequivocal, the question as to the most optimal ECMO centre size remains unanswered. Importantly, since studies looking at the effect of centre size on patient outcomes utilised various definitions of centre sizes, this highlights a need for a consistent approach in defining the case load groups for future analysis. Moreover, there is a need to control for the potential impact of differences in patient morbidity between centres in such studies, since if larger centres receive more complex patients who are at risk of poorer outcomes, this might mask any benefit of receiving care in such large volume centres.

## Conclusions

To our knowledge, this is the first study that provides a summary of the available evidence on ECMO centre size on patient outcomes, the impact of nurse-delivered vs clinical perfusion scientists-delivered bedside ECMO care. While we have highlighted the heterogeneity and limitations of the included non-RCT studies, our review offers the most comprehensive summary of key data relating to the clinical delivery of ECMO services to date.

The findings presented in our review are particularly pertinent to centres which hope to deliver a respiratory ECMO programme alongside a cardiac ECMO programme. Institutions considering the implementation of an ECMO service should conduct their own cost analysis, accounting for labour costs, predicted ECMO utilisation, local staff availability, and the impact of utilising clinical perfusion scientists in ECMO care on cardiac surgery services. Finally, when choosing a model of ECMO delivery, costs should be regarded as an important consideration, and this should include the costs that families will incur when visiting patients.

Funding statement: This work was supported by Welsh Health Specialised Services Committee (now subsumed into the NHS Wales Joint Commissioning Committee), who commissioned the original evidence review.

Competing interest: Andrew Champion and Iolo Doull were employees of Welsh Health Specialised Services Committee when this study was conducted. No other conflicts of interest exist.

## Supplemental Material

Supplemental Material - ECMO for Adult Respiratory Failure: A Rapid Review of Clinical and Service Delivery Evidence to Guide Policy in Wales Supplemental Material for ECMO for Adult Respiratory Failure: A Rapid Review of Clinical and Service Delivery Evidence to Guide Policy in Wales by Michal Pruski, Michael Beddard, Susan O’Connell, Andrew Champion, Rhys Morris, Richard Pugh, and Iolo Doull in Seminars in Cardiothoracic and Vascular Anesthesia

Supplemental Material - ECMO for Adult Respiratory Failure: A Rapid Review of Clinical and Service Delivery Evidence to Guide Policy in Wales Supplemental Material for ECMO for Adult Respiratory Failure: A Rapid Review of Clinical and Service Delivery Evidence to Guide Policy in Wales by Michal Pruski, Michael Beddard, Susan O’Connell, Andrew Champion, Rhys Morris, Richard Pugh, and Iolo Doull in Seminars in Cardiothoracic and Vascular Anesthesia

Supplemental Material - ECMO for Adult Respiratory Failure: A Rapid Review of Clinical and Service Delivery Evidence to Guide Policy in Wales Supplemental Material for ECMO for Adult Respiratory Failure: A Rapid Review of Clinical and Service Delivery Evidence to Guide Policy in Wales by Michal Pruski, Michael Beddard, Susan O’Connell, Andrew Champion, Rhys Morris, Richard Pugh, and Iolo Doull in Seminars in Cardiothoracic and Vascular Anesthesia

Supplemental Material - ECMO for Adult Respiratory Failure: A Rapid Review of Clinical and Service Delivery Evidence to Guide Policy in Wales Supplemental Material for ECMO for Adult Respiratory Failure: A Rapid Review of Clinical and Service Delivery Evidence to Guide Policy in Wales by Michal Pruski, Michael Beddard, Susan O’Connell, Andrew Champion, Rhys Morris, Richard Pugh, and Iolo Doull in Seminars in Cardiothoracic and Vascular Anesthesia
