# Supplementary material for: ECMO for Adult Respiratory Failure: A Rapid Review of Clinical and Service Delivery Evidence to Guide Policy in Wales
Source: Semin Cardiothorac Vasc Anesth. 2024 Dec 22;29(3):192–202. doi: 10.1177/10892532241309787 (PMC12340146; doi:10.1177/10892532241309787)
Supplement: Supplemental Material - ECMO for Adult Respiratory Failure: A Rapid Review of Clinical and Service Delivery Evidence to Guide Policy in Wales [file sj-pdf-1-scv-10.1177_10892532241309787.pdf]

Supplementary File 1 – Medline, Embase and Cochrane Library search strategies (ID, search terms, search hits)

Ovid MEDLINE(R) ALL <1946 to October 06, 2021>

1 respiratory insufficiency/ or acidosis, respiratory/ or hypoventilation/ 37825

2 Hypoxia/ 67379

3 Hypercapnia/ 8888

4 respiratory failure.tw. 34877

5 (acute respiratory distress syndrome or ARDS).tw. 23119

6 (hypox* or hyperca*).tw. 210516

7 1 or 2 or 3 or 4 or 5 or 6 301710

8 (ECMO or ECLS).tw. 10421

9 ((extracorporeal or extra corporeal) adj membrane oxygenation).tw. 13198

10 ((extracorporeal or extra corporeal) adj Life Support).tw. 2288

11 Extracorporeal Membrane Oxygenation/ 12613

12 8 or 9 or 10 or 11 19313

13 7 and 12 5541

14 randomized controlled trial.pt. 545754

15 controlled clinical trial.pt. 94447

16 randomized.ab. 536269

17 placebo.ab. 222020

18 drug therapy.fs. 2382884

19 randomly.ab. 367240

20 trial.ab. 570906

21 groups.ab. 2255653

22 14 or 15 or 16 or 17 or 18 or 19 or 20 or 21 5137768

23 exp animals/ not humans.sh. 4895145

24 22 not 23 4469329

25 13 and 24 977

26 *"Models, Organizational"/ 6459

27 (service delivery or service evaluation).tw. 16292

28 (ECMO adj2 (program or model)).tw. 157

29 (perfusionist* or (ECMO adj2 specialist*)).tw. 582

30 staff* model*.tw. 899

31 or/27-30 17886

32 12 and 31 248

33 25 or 32 1213

34 limit 33 to (english language and yr="2000 -Current") 952

Embase Classic+Embase <1947 to 2021 October 06>

1 respiratory failure/ 83773

2 respiratory acidosis/ 6803

3 hypoventilation/ 7883

4 hypoxia/ 122335

5 hypercapnia/ 22898

6 respiratory failure.tw. 60434

7 (acute respiratory distress syndrome or ARDS).tw.

8 (hypox* or hyperca*).tw. 302975

9 1 or 2 or 3 or 4 or 5 or 6 or 7 or 8 461808

10 (ECMO or ECLS).tw. 21378

34688

11 ((extracorporeal or extra corporeal) adj membrane oxygenation).tw. 19447

12 ((extracorporeal or extra corporeal) adj Life Support).tw. 3600

13 extracorporeal oxygenation/ 28659

14 10 or 11 or 12 or 13 38993

15 9 and 14 10944

16 (random$ or factorial$ or crossover$ or cross over$ or cross-over$ or placebo$ or (doubl$

adj blind$) or (singl$ adj blind$) or assign$ or allocat$ or volunteer$).tw. 2491567

17 crossover-procedure/ 68668

18 double-blind procedure/ 190999
19 randomized controlled trial/ 681347
20 single-blind procedure/ 43974

21 16 or 17 or 18 or 19 or 20 2597723

22 15 and 21 702

23 *"Models, Organizational"/ 4012

24 (service delivery or service evaluation).tw. 22326

25 (ECMO adj2 (program or model)).tw. 356

26 (perfusionist* or (ECMO adj2 specialist*)).tw. 1045

27 staff* model*.tw. 1272

28 23 or 24 or 25 or 26 or 27 28823

29 14 and 28 623

30 22 or 29 1314

31 limit 30 to (english language and yr="2000 -Current") 1144

Cochrane Library

#1 MeSH descriptor: [Respiratory Insufficiency] explode all trees 2959

#2 MeSH descriptor: [Hypoventilation] explode all trees 137

#3 MeSH descriptor: [Acidosis, Respiratory] explode all trees 50

#4 MeSH descriptor: [Hypoxia] explode all trees 2205

#5 MeSH descriptor: [Hypercapnia] explode all trees 511

#6 (acute respiratory distress syndrome or ARDS):ti,ab,kw (Word variations have been searched) 3646

#7 (respiratory failure):ti,ab,kw (Word variations have been searched) 9819

#8 ((acute respiratory distress syndrome or ARDS)):ti,ab,kw (Word variations have been searched) 3646

#9 ((hypox* or hyperca*)):ti,ab,kw (Word variations have been searched) 14035

#10 #1 OR #2 OR #3 OR #4 OR #5 OR #6 OR #7 OR #8 OR #9 26221

#11 (ECMO or ECLS):ti,ab,kw (Word variations have been searched) 899

#12 (((extracorporeal or extra corporeal) NEAR Life Support)):ti,ab,kw (Word variations have been searched) 86

#13 (((extracorporeal or extra corporeal) NEAR membrane oxygenation)):ti,ab,kw (Word variations have been searched) 744

#14 MeSH descriptor: [Extracorporeal Membrane Oxygenation] explode all trees 191

#15 #11 OR #12 OR #13 OR #14 1242

#16 #10 AND #15 398

#17 MeSH descriptor: [Models, Organizational] explode all trees 176

#18 (service delivery or service evaluation):ti,ab,kw (Word variations have been searched) 26285

#19 ((ECMO NEAR/2 (program or model))):ti,ab,kw (Word variations have been searched) 8

#20 ((perfusionist* or (ECMO NEAR/2 specialist*))):ti,ab,kw (Word variations have been searched) 52

#21 (staff* model*):ti,ab,kw (Word variations have been searched) 3560

#22 #17 OR #18 OR #19 OR #20 OR #21 29146

#23 #15 AND #22 48

#24 #23 or #16 with Cochrane Library publication date Between Jan 2000 and Sep 2021, in Cochrane Reviews 7

#25 #23 OR #16 with Publication Year from 2000 to 2021, in Trials (Word variations have been searched) 369

#26 #24 OR #25 376
