# Supplementary material for: ECMO for Adult Respiratory Failure: A Rapid Review of Clinical and Service Delivery Evidence to Guide Policy in Wales
Source: Semin Cardiothorac Vasc Anesth. 2024 Dec 22;29(3):192–202. doi: 10.1177/10892532241309787 (PMC12340146; doi:10.1177/10892532241309787)
Supplement: Supplemental Material - ECMO for Adult Respiratory Failure: A Rapid Review of Clinical and Service Delivery Evidence to Guide Policy in Wales [file sj-pdf-2-scv-10.1177_10892532241309787.pdf]

**Population**	Adult patients with respiratory failure, including those who cannot be mechanically ventilated due to e.g. tracheal injury
**Intervention**	• Extracorporeal Membrane Oxygenation (+/- any low volume ventilation with appropriate adjuncts).<br>• Nurse delivered Extracorporeal Membrane Oxygenation<br>• Extracorporeal Membrane Oxygenation delivered in a high-volume centre (note that centres that deliver both cardiac and respiratory ECMO were included – it is the total number of ECMO patients that count towards centre size, though outcomes of interest only concern the respiratory ECMO patients)
**Comparison**	• Mechanical Ventilation +/- relevant ventilation adjuncts (e.g. nitric oxide, oscillation, ECCOR) and those aimed at managing blood pH.<br>• Perfusionist delivered Extracorporeal Membrane Oxygenation<br>• Extracorporeal Membrane Oxygenation delivered in a low-volume centre (see note above)
**Outcomes**	• Mortality<br>• Morbidity<br>• Length of stay (hospital and critical care)<br>• Survival length to relevant time point (e.g. transplant)<br>• Overall survival<br>• Quality of life costs<br>• Adverse events<br>• Treatment duration<br>• Distance travelled to receive the intervention.
**Limits**	• English language only<br>• Studies not involving lung protective ventilation<br>• Studies published after 2000
**Types of evidence included**	Systematic reviews and meta-analyses and reports of primary studies that have been published since an included review.
**Exclusions**	Conference abstracts, case reports, non-systematic reviews, editorials, letters.

Supplementary File 2. Inclusion and exclusion criteria for this rapid evidence review.
