# Supplementary material for: ECMO for Adult Respiratory Failure: A Rapid Review of Clinical and Service Delivery Evidence to Guide Policy in Wales
Source: Semin Cardiothorac Vasc Anesth. 2024 Dec 22;29(3):192–202. doi: 10.1177/10892532241309787 (PMC12340146; doi:10.1177/10892532241309787)
Supplement: Supplemental Material - ECMO for Adult Respiratory Failure: A Rapid Review of Clinical and Service Delivery Evidence to Guide Policy in Wales [file sj-pdf-3-scv-10.1177_10892532241309787.pdf]

Supplementary File 3 – Details of excluded studies

First Author	Year	Title	Reason for exclusion
Ahmed	2021	Outcomes of Extracorporeal Membrane Oxygenation in Acute Respiratory Distress Syndrome Following Traumatic Injury: A Propensity-Matched Analysis	Clinical, not RCT
Alshahrani	2018	Extracorporeal membrane oxygenation for severe Middle East respiratory syndrome coronavirus	Clinical, not RCT
Combes	2018	Extracorporeal membrane oxygenation for severe acute respiratory distress syndrome	RCT included in a meta-analysis
Daly	2017	An international survey: the role of specialist nurses in adult respiratory extracorporeal membrane oxygenation	Not in scope
Fichtner	2018	Mechanical Ventilation and Extracorporeal Membrane Oxygena tion in Acute Respiratory Insufficiency	Not in scope
Goligher	2018	Extracorporeal Membrane Oxygenation for Severe Acute Respiratory Distress Syndrome and Posterior Probability of Mortality Benefit in a Post Hoc Bayesian Analysis of a Randomized Clinical Trial	RCT included in a meta-analysis
Grasselli	2019	Quality of Life and Lung Function in Survivors of Extracorporeal Membrane Oxygenation for Acute Respiratory Distress Syndrome	Clinical, not RCT
Kolaitis	2018	Improvement in patient-reported outcomes after lung transplantation is not impacted by the use of extracorporeal membrane oxygenation as a bridge to transplantation	Not in scope
Kurniawati	2021	Quality of life following adult veno-venous extracorporeal membrane oxygenation for acute respiratory distress syndrome: a systematic review	Clinical systematic review including non-RCTs
Li	2021	Extracorporeal membrane oxygenation (ECMO) for critically ill patients with coronavirus disease 2019 (COVID-19): A retrospective cohort study	Clinical, not RCT
Linke	2020	A survey of extracorporeal membrane oxygenation practice in 23 Australian adult intensive care units	Not in scope
Mendes	2019	Extracorporeal membrane oxygenation for severe acute respiratory distress syndrome in adult patients: a systematic review and meta-analysis	Other clinical meta-analysis; same studies as Combes et al., but not patient level data.
Mitchell	2010	A systematic review to inform institutional decisions about the use of extracorporeal membrane oxygenation during the H1N1 influenza pandemic	Not in scope
Mongero	2013	Managing the extracorporeal membrane oxygenation (ECMO) circuit integrity and	Not in scope

		safety utilizing the perfusionist as the "ECMO Specialist	
Munshi	2014	Extracorporeal life support for acute respiratory failure. A systematic review and metaanalysis	Other clinical meta-analysis; not up-to-date
Oude Lansink-Hartgring	2016	Hospital Costs Of Extracorporeal Life Support Therapy	Not in scope
Oude Lansink-Hartgring	2021	Hospital Costs of Extracorporeal Membrane Oxygenation in Adults: A Systematic Review	Not in scope
Rinieri	2015	National review of use of extracorporeal membrane oxygenation as respiratory support in thoracic surgery excluding lung transplantation	Not in scope
Schechter	2016	Spontaneously Breathing Extracorporeal Membrane Oxygenation Support Provides the Optimal Bridge to Lung Transplantation	Not in scope
Shaefi	2021	Extracorporeal membrane oxygenation in patients with severe respiratory failure from COVID-19	Clinical, not RCT
Sud	2021	Comparative Effectiveness of Protective Ventilation Strategies for Moderate and Severe Acute Respiratory Distress Syndrome. A Network Meta-Analysis	Other clinical meta-analysis; does not match the scope as well as Aoyama et al.
Sylvestre	2019	Long-term neurocognitive outcome is not worsened by of the use of venovenous ECMO in severe ARDS patients	Clinical, not RCT
Todd	2017	Extracorporeal membrane oxygenation as a bridge to lung transplantation: A single-center experience in the present era	Not in scope
Tseng	2011	Costs Associated with Extracorporeal Life Support Used in Adults: A Single-Center Study	Cost study with no comparator
Wilcox	2020	Long-Term Quality of Life After Extracorporeal Membrane Oxygenation in ARDS Survivors: Systematic Review and Meta-Analysis	Clinical systematic review including non-RCTs
Zhu	2021	Extracorporeal membrane oxygenation versus mechanical ventilation alone in adults with severe acute respiratory distress syndrome: A systematic review and meta-analysis	Other clinical meta-analysis; includes observational studies
