# Supplementary material for: ECMO for Adult Respiratory Failure: A Rapid Review of Clinical and Service Delivery Evidence to Guide Policy in Wales
Source: Semin Cardiothorac Vasc Anesth. 2024 Dec 22;29(3):192–202. doi: 10.1177/10892532241309787 (PMC12340146; doi:10.1177/10892532241309787)
Supplement: Supplemental Material - ECMO for Adult Respiratory Failure: A Rapid Review of Clinical and Service Delivery Evidence to Guide Policy in Wales [file sj-pdf-4-scv-10.1177_10892532241309787.pdf]

Supplementary File 4 – Details of the included clinical studies

Study	Study and patient Inclusion Criteria	Patient number	Outcomes	Sub-analysis	Study Limitations	Context
Aoyama et al. 2019[18]	RCTs or quasi-RCTs<br><br>Adult patients with moderate to severe ARDS who received mechanical ventilation in ICU	Total = 7753<br><br>Patients within individual comparisons:<br>Veno-venous ECMO vs LPV = 429 (same two studies as in Combers et al. 2020)<br>LPV vs HFOV = 1403<br>LPV vs NMBA = 1461<br>LPV vs Open lung strategy = 3550<br>INO and RM vs Open lung strategy = 17<br>INO and EM vs INO = 17<br>INO vs Open lung strategy = 12<br>HFOV vs Prone positioning = 26<br>HFOV vs HFOV and Prone positioning = 26<br>HFOV and Prone positioning vs Prone positioning = 26	28-Day Mortality [Network Risk Ratio (95% CrI)]:<br>LPV vs ECMO 0.60 (0.38 - 0.93; favours ECMO)<br>ECMO vs HFOV 1.88 (1.12 - 3.24; indirect evidence; favours ECMO)<br><br>Barotrauma (at any time point): no statistically significant ECMO comparisons	N/A	As assessed by TSD7: only ordinal values have been given for the assessment of the heterogeneity in the relative treatment effects; there is no justification given for the use of random models; there is no discussion of the statistical heterogeneity between baseline arms; no mention of information pertaining to the baseline model; no mention of baseline and relative effects estimation justification, nor of the studies used to inform them; no value given for the number of potential inconsistencies in the network.	World-wide studies, covering a wide array of interventions.
Combes et al. 2020[21]	RCTs evaluating veno-venous ECMO in the experimental group and conventional ventilatory management in the control group; note that two studies were	ECMO = 214<br>Control =215	Mortality [relative risk (95% CI)]: 90-Day 0.75 (0.6–0.94; favours ECMO)<br>60-Day 0.73 (0.58–0.92; favours ECMO)	Sub-analysis on the primary outcome (90-Day Mortality):<br>Significant difference between patients with 1-2 organs failed vs 3 or more organs failed (p=0.006), with the former group responding better to ECMO	As assessed by AMSTAR-2: no justification for excluded individual studies; no assessment of presence and likely impact of publication bias; the justification for the meta-analysis is only implicit; there was no explanation for only including RCTs;	Two RCTs studies included, one involving primarily French centres, but also centres forom the USA, Canada and Australia, the other was a UK trial. Both trials had a pragmatic

	excluded because they were over 15 years old.<br><br>Adult patients with ARDS fulfilling the American–European Consensus Conference definition or the Berlin definition for ARDS.		28-Day 0.57 (0.4–0.81; favours ECMO)<br><br>90-Day treatment failure [relative risk (95% CI)] 0.65 (0.52–0.8; favours ECMO)<br><br>Other outcome measures given, but with no indication of significance.	No significant difference between: Female and male patients<br>Under 49 years old and those 49 years or older<br>With a primary diagnosis of pneumonia vs other<br>Interval between start of mechanical ventilation and randomisation being up to or longer than 3 days<br>PaO_2/FiO_2 being up to or over 68 at randomisation<br>PEEP being up to or over 12 at randomisation<br>Murray score being up to 3.3 or over at randomisation<br>Predicted mortality being up to or over 0.3	some detail was missing from the description of included studies; there was insufficient detail provided in the conflict of interest section.<br><br>While many sub-analyses have been undertaken. The study might not be powered to derived reliable conclusions from these sub-analyses.	element to them in that one allowed for adjunctive therapies, such as inhaled nitric oxide, recruitment manoeuvres, HFOV, or almitrine infusion, while the other study only advised to follow a low-volume low-pressure ventilation strategy.
